# Effectiveness of Exercise and Physiotherapy in Chemotherapy-Induced Peripheral Neuropathy: A Systematic Review

**DOI:** 10.3390/healthcare13222973

**Published:** 2025-11-19

**Authors:** Javier Antonio Tamayo Fajardo, Francisco León Parejo

**Affiliations:** 1 Department of Health Sciences, Faculty of Nursing, Universidad de Huelva, 21007 Huelva, Spain; 2 Department of Health Sciences, Universidad Internacional de Andalucía (UNIA), 41092 Sevilla, Spain; francisco.leonparejo@estudiante.unia.es

**Keywords:** chemotherapy-induced peripheral neuropathy, exercise, physiotherapy, exercise therapy, cancer rehabilitation, quality of life

## Abstract

**Background:** Chemotherapy-induced peripheral neuropathy (CIPN) is a common and disabling adverse effect of cancer treatment, affecting up to 65% of patients. It reduces quality of life, increases fall risk, and often leads to chemotherapy dose reduction or discontinuation. Because pharmacological management provides limited relief, non-pharmacological strategies such as exercise and physiotherapy have become increasingly relevant. **Methods:** A systematic review following the PRISMA 2020 guidelines was conducted to identify randomised controlled trials (RCTs) evaluating exercise and physiotherapy for the prevention or treatment of CIPN. PubMed, Scopus, Web of Science, and Cochrane Library were searched up to May 2025. Methodological quality was assessed with the PEDro scale, and due to heterogeneity, a narrative synthesis was performed. Outcomes included neuropathic symptoms, pain, motor and sensory function, balance, muscle strength, and quality of life. **Results:** Twenty-six RCTs published between 2017 and 2025 were included. Nineteen assessed exercise-based interventions (aerobic, resistance, sensorimotor, balance, yoga, or multimodal), and seven examined physiotherapy modalities (manual therapy, photobiomodulation, Scrambler therapy, ultrasound, or electrical stimulation). Both approaches improved sensory and motor symptoms, balance, muscle strength, and quality of life. Adherence ranged from 70% to 95%, and no serious adverse events were reported. However, variability in intervention design and outcome measures precluded meta-analysis. **Conclusions:** Exercise and physiotherapy are safe, feasible, and effective non-pharmacological strategies for managing CIPN. However, heterogeneity in intervention design highlights the need for high-quality RCTs to establish optimal protocols and standardised clinical guidelines.

## 1. Introduction

Chemotherapy-induced peripheral neuropathy (CIPN) is one of the most prevalent and disabling adverse effects of systemic anticancer therapy, with reported incidence rates ranging from 30% to 65%, depending on the neurotoxic agent and cumulative dose [1,2]. CIPN frequently leads to chemotherapy dose modification or discontinuation, compromising treatment efficacy and overall survival [3,4]. Common symptoms include paraesthesia, numbness, neuropathic pain, balance impairment, and reduced muscle strength, which collectively limit patients’ functional independence and quality of life [5,6].

The pathophysiology of CIPN involves axonal degeneration, mitochondrial dysfunction, oxidative stress, altered ion channel activity, and neuroinflammation [7,8]. Pharmacological options such as duloxetine or gabapentinoids provide only partial symptom relief and are often limited by adverse effects [9]. Therefore, non-pharmacological strategies—particularly exercise and physiotherapy—have gained growing attention as complementary approaches.

Exercise exerts neuroprotective, anti-inflammatory, and antioxidant effects, promoting mitochondrial biogenesis, axonal regeneration, and neuromuscular function [10,11]. Similarly, physiotherapy modalities including manual therapy, neuromotor stimulation, and photobiomodulation can enhance proprioception, reduce pain, and improve functional performance [12,13,14].

Despite emerging evidence, methodological heterogeneity and variability in intervention design persist, making it difficult to establish standardised protocols [15]. Such inconsistencies highlight the need for a comprehensive synthesis of randomised controlled trials to clarify the effectiveness of exercise and physiotherapy in chemotherapy-induced peripheral neuropathy (CIPN). This systematic review therefore aims to synthesise current evidence to guide clinical decision-making and the development of standardised exercise and physiotherapy protocols for patients undergoing neurotoxic chemotherapy.

## 2. Materials and Methods

A systematic review was conducted to identify randomised controlled trials (RCTs) examining the effects of exercise and physiotherapy on chemotherapy-induced peripheral neuropathy (CIPN). The review followed the Preferred Reporting Items for Systematic Reviews and Meta-Analyses (PRISMA) 2020 guidelines and PRISMA 2020 Checklist to ensure transparency and reproducibility [16]. The protocol for this review was retrospectively registered in the Open Science Framework (OSF; registration DOI: https://doi.org/10.17605/OSF.IO/5YJZW).

### 2.1. Search Strategy

Electronic searches were performed in PubMed, Scopus, Web of Science, and the Cochrane Library from inception to May 2025, complemented by manual searches of reference lists (Table 1). No additional records were identified through manual reference screening.

The search strategy combined MeSH terms and free-text keywords using Boolean operators: (“chemotherapy-induced peripheral neuropathy” OR “CIPN”) AND (“exercise” OR “training” OR “physiotherapy” OR “physical therapy”) AND (“cancer” OR “oncology”).

Filters and limits applied included: Humans, Randomised Controlled Trial, English language, and publication years 2015–2025.

The number of records retrieved from each database was as follows: PubMed (n = 38), Scopus (n = 202), Web of Science (n = 291), and Cochrane Library (n = 91), for a total of 622 records before removing duplicates.

### 2.2. Eligibility Criteria

Studies were included if they met the following criteria:

- Randomised controlled trials involving adult patients receiving neurotoxic chemotherapy (taxanes, platinum agents, vinca alkaloids, or proteasome inhibitors).
- Interventions consisting of exercise or physiotherapy, compared with usual care or control.
- Outcomes including CIPN incidence or severity, neuropathic pain, motor and sensory function, balance, muscle strength, or quality of life.

Studies were excluded if they were non-randomised, pharmacological-only trials, preclinical investigations, or paediatric populations.

For synthesis purposes, studies were grouped according to the primary type of intervention: exercise-based programmes and physiotherapy-based modalities. Physiotherapy-based modalities included interventions such as photobiomodulation, manual therapy, Scrambler therapy, transcutaneous electrical nerve stimulation (TENS), and therapeutic ultrasound.

### 2.3. Selection Process and Data Collection

**Figure 1:**
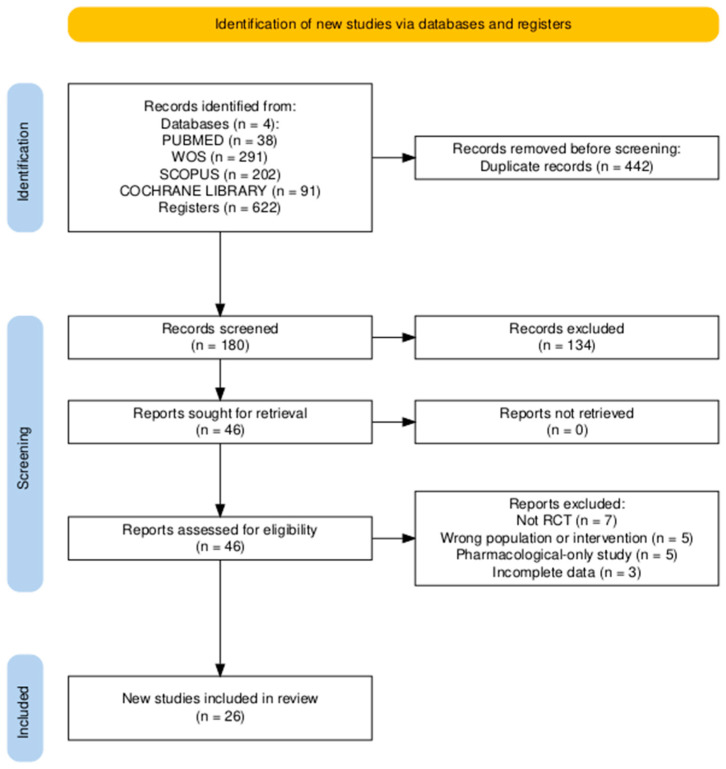
PRISMA 2020 flow diagram showing the study selection process.

Titles and abstracts were independently screened by two reviewers (J.A.T.F. and F.L.P.), and potentially eligible full texts were assessed according to the predefined inclusion and exclusion criteria. Data extraction from the included studies was also performed independently by the same reviewers using a predefined data extraction form in Microsoft Excel. Extracted information included authorship, publication year, study design, sample characteristics, intervention type, duration, frequency, outcome measures, main findings, adherence, adverse events, and PEDro score. When specific data were missing or unclear in the original reports, they were recorded as “not reported” (NR) and not imputed. Any discrepancies during study selection or data extraction were resolved through discussion and consensus. No automation tools or machine learning algorithms were used at any stage of the process. The study selection procedure is illustrated in the PRISMA 2020 flow diagram (Figure 1).

### 2.4. Methodological Quality

The methodological quality of the included RCTs was assessed using the Physiotherapy Evidence Database (PEDro) scale [17], which rates studies based on 11 items related to internal validity and interpretability. Scores range from 0 to 10, with higher scores indicating better quality. A score ≥6 was considered high quality. Two reviewers (J.A.T.F. and F.L.P.) independently assessed each study, and disagreements were resolved by consensus.

For each outcome, effect measures were extracted as reported in the original studies, typically including mean values, standard deviations, mean differences, percentage changes, and *p*-values for between-group comparisons. Although many included studies were of moderate-to-high methodological quality, a quantitative meta-analysis was not considered appropriate due to substantial heterogeneity across trials. This heterogeneity involved differences in intervention characteristics (e.g., exercise type, physiotherapy modality, duration, and supervision), outcome measures (e.g., EORTC QLQ-CIPN20, FACT/GOG-NTX, Total Neuropathy Score, or CIPNAT), timing of assessments (during vs. after chemotherapy), and incomplete reporting of variance data in several studies. Therefore, a structured narrative synthesis was performed following the PRISMA 2020 guidance for heterogeneous interventions.

Risk of publication bias was not formally assessed, as no meta-analysis was conducted. However, all available randomised controlled trials meeting the inclusion criteria were included to minimise selection and reporting bias. The overall certainty of evidence was assessed qualitatively, considering study quality, consistency of results, and sample sizes.

## 3. Results

The selection and inclusion process is summarised in the PRISMA 2020 flow diagram (Figure 1), and the characteristics of the included studies are presented in Table 2.

A total of 622 records were identified through database searches (PubMed, Scopus, Web of Science, and Cochrane Library). After removing 442 duplicates, 180 unique records remained for title and abstract screening. Of these, 134 records were excluded at the title and abstract screening stage, did not meet the inclusion criteria in terms of population, intervention, or study design, and 46 full-text articles were assessed for eligibility. Finally, 26 randomised controlled trials (RCTs) met the inclusion criteria and were included in the qualitative synthesis.

### 3.1. General Characteristics of the Included Studies

The 26 studies, published between 2017 and 2025, involved approximately 1650 participants receiving neurotoxic chemotherapy. Most trials were conducted in Europe, Asia, and North America using parallel-group RCT designs. Intervention duration ranged from 6 to 24 weeks, with 2–5 sessions per week. Average adherence ranged from 70% to 95%, and no severe adverse events were reported.

The main characteristics and findings of the included studies are summarised in Table 2. Most trials compared an exercise-based or physiotherapy-based intervention with usual care or non-exercise control conditions. The majority reported significant improvements in sensory and motor symptoms, balance, muscle strength, and overall quality of life in participants undergoing neurotoxic chemotherapy. Aerobic, resistance, and sensorimotor exercise programmes were particularly effective in reducing neuropathic symptoms and improving functional performance, while physiotherapy-based modalities such as photobiomodulation, transcutaneous electrical nerve stimulation (TENS), Scrambler therapy, manual therapy, and therapeutic ultrasound also showed positive effects on pain reduction and sensory recovery. No serious adverse events were reported in any study.

### 3.2. Exercise-Based Interventions

Nineteen studies implemented structured exercise programmes during or after chemotherapy. Common modalities included combined aerobic and resistance training [19,21,38], sensorimotor and balance exercises [32,39], yoga and low-intensity routines [23,35], and multimodal or home-based interventions [22,30].

Assessment tools included EORTC QLQ-CIPN20, FACT/GOG-NTX, Total Neuropathy Score (TNS), SPPB, Six-Minute Walk Test (6MWT), and handgrip dynamometry.

Overall, exercise interventions significantly reduced the sensory and motor symptoms, improved balance, muscle strength, and functional performance, and enhanced quality of life. Preventive effects were reported when exercise was initiated early during chemotherapy [27,31].

### 3.3. Physiotherapy-Based Interventions

Seven RCTs investigated physiotherapy-based modalities, including photobiomodulation, manual therapy, Scrambler therapy, transcutaneous electrical nerve stimulation (TENS), and therapeutic ultrasound. Studies by Joy et al. [12] and Sassmann et al. [13] demonstrated significant improvements in sensory symptoms, neuropathic pain, and functional capacity, with enhanced EORTC QLQ-CIPN20 scores. Photobiomodulation effectively prevented the progression of taxane-induced neuropathy, while Scrambler therapy and TENS produced benefits in chronic CIPN. No major adverse effects were reported, confirming the safety and tolerability of physiotherapy-based interventions.

### 3.4. Risk of Bias Assessment

PEDro scale scores ranged from 6 to 10 out of 10, with a mean score of 8.2, indicating overall high methodological quality among the included RCTs (see Table 2 for individual scores). Randomisation and baseline comparability were generally adequate, and most studies clearly reported between-group comparisons and measures of variability. However, allocation concealment and blinding of participants and therapists remained limited due to the nature of physical and exercise-based interventions.

### 3.5. Synthesis of Results

Both exercise and physiotherapy interventions were effective, safe, and feasible for the management of chemotherapy-induced peripheral neuropathy (CIPN). The most consistent outcomes across trials were reductions in neuropathic symptoms and improvements in balance, muscle strength, and quality of life. The overall findings were coherent among studies of high methodological quality according to the PEDro scale. Due to the heterogeneity of interventions, assessment tools, and follow-up durations, a narrative synthesis was performed rather than a quantitative meta-analysis.

### 3.6. Reporting Biases

Publication bias was not formally assessed, as no quantitative meta-analysis was performed. Nevertheless, all eligible randomised controlled trials that met the inclusion criteria were incorporated into the synthesis to minimise the risk of selective reporting bias. No evidence of outcome reporting discrepancies was detected among the included studies.

## 4. Discussion

This systematic review synthesised evidence from 26 randomised controlled trials evaluating the effects of exercise and physiotherapy on chemotherapy-induced peripheral neuropathy (CIPN). The findings consistently demonstrated that both approaches are safe, feasible, and beneficial in alleviating neuropathic symptoms, improving balance and muscle strength, and enhancing the overall quality of life in patients receiving neurotoxic chemotherapy.

These results are consistent with previous systematic reviews and international clinical practice guidelines recommending exercise and rehabilitation as supportive interventions in oncology care. In particular, the ASCO Clinical Practice Guideline and the ESMO–EONS–EANO guideline on therapy-induced neurotoxicity emphasise the role of structured exercise and physical rehabilitation in the management of chemotherapy-induced peripheral neuropathy [3,6]. Likewise, the findings of this review are consistent with previous systematic reviews that also reported improvements in neuropathic symptoms and physical function following structured exercise interventions in patients receiving neurotoxic chemotherapy [3,40]. More recently, Amarelo et al. [41] conducted a meta-analysis confirming that exercise significantly alleviates chemotherapy-induced peripheral neuropathy and enhances quality of life. Together, these findings reinforce the growing evidence supporting exercise and physiotherapy as effective strategies for the prevention and management of CIPN. Multimodal exercise programmes that combine aerobic, resistance, and balance training appear particularly effective, supporting the neuroprotective, anti-inflammatory, and antioxidant effects of regular exercise [26,29]. Early initiation of exercise during chemotherapy, as reported by Kleckner et al. [14] and Streckmann et al. [15], may mitigate nerve toxicity and prevent functional decline.

Physiotherapy-based modalities such as photobiomodulation and transcutaneous electrical stimulation have also shown promising outcomes. Studies by Joy et al. [12] and Sassmann et al. [13] demonstrated significant reductions in neuropathic pain and improvements in sensory function without adverse effects. The potential mechanisms involve enhanced microcirculation, reduced inflammation, and modulation of nociceptive signalling pathways [25,37].

High adherence rates (70–95%) across studies confirm the feasibility of implementing these interventions within oncology settings [25]. Adherence was likely facilitated by professional supervision, individualised programming, and the perceived benefit of symptom relief. Importantly, no severe adverse events were reported, reinforcing the safety profile of both exercise and physiotherapy approaches.

Compared with earlier literature, recent trials demonstrate greater methodological rigour and more diverse intervention designs, evolving from traditional aerobic exercise towards functional, resistance, and sensorimotor training [20]. Similarly, physiotherapy interventions have advanced towards active, integrative modalities that combine manual therapy and neuromuscular re-education, providing broader rehabilitative benefits.

The physiological mechanisms underlying these effects may include improved peripheral nerve perfusion, modulation of oxidative stress and inflammatory mediators, and upregulation of neurotrophic factors such as brain-derived neurotrophic factor (BDNF), which supports axonal regeneration and neural plasticity [28]. Collectively, these findings highlight the potential of physical rehabilitation not only as symptom management but also as a neuroprotective therapeutic strategy.

Despite the encouraging evidence, several limitations should be acknowledged. Considerable heterogeneity exists in intervention type, duration, and intensity, which limits comparability and standardisation. Small sample sizes in some trials may have reduced statistical power and inflated treatment effects. Furthermore, blinding of participants and therapists remains challenging in non-pharmacological interventions. Variability in assessment tools—such as EORTC QLQ-CIPN20, FACT/GOG-NTX, and TNS—also hinders quantitative synthesis.

Another important limitation is the lack of long-term follow-up in most trials, making it difficult to determine the durability of treatment effects. Future research should therefore prioritise multicentre RCTs with longer follow-up periods and standardised intervention protocols. The inclusion of objective biomarkers, such as neurophysiological or inflammatory markers, could further elucidate the mechanisms of neuroprotection and functional recovery. Additionally, this review was limited to studies published in English and may therefore be subject to language or publication bias despite comprehensive database searches.

Furthermore, certain methodological constraints of this review should be acknowledged. The protocol was retrospectively registered in the Open Science Framework (OSF; registration DOI: https://doi.org/10.17605/OSF.IO/5YJZW) to ensure methodological transparency. Grey literature was not systematically searched, which may have led to the omission of unpublished or non-indexed studies. However, multiple databases were comprehensively searched, and a rigorous two-reviewer selection process was employed to minimise bias.

Notwithstanding these limitations, this review possesses notable strengths, including a comprehensive multi-database search strategy, exclusive inclusion of RCTs, and methodological appraisal using the PEDro scale. The incorporation of recent studies up to 2025 enhances the relevance of the findings for current clinical practice.

From a clinical perspective, the evidence supports integrating structured exercise and physiotherapy into standard oncology care. Exercise programmes should be initiated early, adapted to individual tolerance and functional capacity, and supervised by trained professionals. Physiotherapy modalities provide additional benefits by targeting sensory symptoms and pain relief, contributing to greater functional independence, psychological well-being, and quality of life.

Overall, this synthesis underscores the growing evidence that structured exercise and physiotherapy represent safe, feasible, and effective components of supportive cancer rehabilitation. Their integration into routine oncology care may help reduce the burden of chemotherapy-induced neuropathy and improve patient outcomes.

Taken together, the consistency of results, high methodological quality of the included RCTs, and absence of major adverse events support a moderate-to-high level of certainty in the evidence that exercise and physiotherapy are effective and safe approaches for managing CIPN.

Future studies should also evaluate cost-effectiveness and implementation strategies to facilitate the integration of these interventions into routine oncology care.

## 5. Conclusions

This systematic review demonstrates that both exercise and physiotherapy are safe, feasible, and effective interventions for the prevention and management of chemotherapy-induced peripheral neuropathy (CIPN). Programmes combining aerobic, resistance, and sensorimotor training consistently improved sensory and motor symptoms, balance, muscle strength, and overall quality of life in patients undergoing neurotoxic chemotherapy.

Physiotherapy modalities such as photobiomodulation and transcutaneous electrical stimulation also produced clinically meaningful benefits without adverse effects, confirming their feasibility as complementary strategies within comprehensive oncology rehabilitation programmes.

Although variability in intervention design and outcome measures limits direct comparison, the overall evidence supports the systematic incorporation of structured exercise and physiotherapy into cancer care. Future research should aim to establish standardised and reproducible protocols, determine optimal therapeutic exercise doses, incorporate objective neurophysiological and biochemical markers, and extend follow-up periods to confirm the durability of observed effects.

In conclusion, exercise and physiotherapy represent key non-pharmacological approaches for improving function, reducing neuropathic symptoms, and enhancing quality of life in individuals affected by chemotherapy-induced peripheral neuropathy. These findings provide a moderate-to-high level of confidence supporting their integration into routine oncology rehabilitation practice.

## Figures and Tables

**Table 1:** Search Strategy.

Database	Search	Filters/Limits	Date of Last Search
PubMed	(“chemotherapy-induced peripheral neuropathy” [MeSH Terms] OR “CIPN” OR “peripheral neuropathy secondary to chemotherapy”) AND (“exercise” OR “training” OR “physiotherapy” OR “physical therapy”) AND (“cancer” OR “oncology” OR “neoplasm”)	Humans, Randomised Controlled Trial, English, Publication years 2015–2025	May 2025
Scopus	TITLE-ABS-KEY (“chemotherapy-induced peripheral neuropathy” OR “CIPN”) AND TITLE-ABS-KEY (“exercise” OR “physiotherapy” OR “training” OR “physical therapy”) AND TITLE-ABS-KEY (“cancer” OR “oncology”)	Article type: randomised controlled trial; Language: English	May 2025
Web of Science	(“chemotherapy-induced peripheral neuropathy” OR “CIPN”) AND (“exercise” OR “physiotherapy” OR “training” OR “physical therapy”) AND (“cancer” OR “oncology”)	Document type: Article; Language: English	May 2025
Cochrane Library	(“chemotherapy-induced peripheral neuropathy” OR “CIPN”) AND (“exercise” OR “training” OR “physiotherapy” OR “physical therapy”) AND (“cancer” OR “oncology” OR “neoplasm”)	Trials; Humans; English; Publication years 2015–2025	May 2025

**Table 2:** Included studies and main characteristics.

Main Author (Year)	Sample and Groups (Number of Participants)	Programme Duration (Frequency)	Intensity	Measurement Instruments	Main Outcomes	Adherence	Adverse Effects	PEDro Score
Al Onazi et al. (2021) [18]	Therapeutic ultrasound (n = 16) vs. Control (n = 15)	2 weeks (10 ultrasound sessions) + 6 weeks of home-based exercise	Continuous ultrasound, 0.7–0.8 W/cm^2^, 3 MHz	FACT/GOG-NTX, QLQ-CIPN20, Semmes-Weinstein monofilament, 128 Hz vibration, Achilles tendon reflex, balance	Significant improvement in symptoms at 2 weeks (*p* = 0.003), with no differences at 6 weeks.	100%	None	8/10
Andersen-Hammond et al. (2020) [11]	Physiotherapy (n = 22) vs. Control (n = 26)	Four sessions with a physiotherapist + daily home exercises	Moderate intensity	Numeric Pain Rating Scale (NPRS), DASH, S-LANSS, Pressure Pain Threshold Test, Handgrip Dynamometry, Quantitative Sensory Testing (QST)	Reduction in neuropathic pain, improved handgrip strength and pain pressure threshold	Not reported (NR)	None	10/10
Argenta et al. (2017) [19]	Photobiomodulation (PBM) (n = 30) vs. Sham (n = 40) + crossover to PBM/PT	6 weeks (PBM 3 sessions per week)	Not applicable (Class IV laser photobiomodulation)	Modified Total Neuropathy Score (mTNS), adherence log	Significant reduction in neuropathic symptoms (−52.6% in mTNS, *p* < 0.001)	>95%	None	10/10
Bao et al. (2020) [20]	Yoga (n = 21) vs. Usual care (n = 20)	8 weeks (2 supervised sessions per week + 5 home-based practices per week)	Moderate intensity	Numeric Rating Scale (NRS), FACT/GOG-Ntx, Functional Reach Test, Chair Stand Test, 4-Meter Gait Speed Test	Improvement in chemotherapy-induced neuropathic pain and functional performance (*p* = 0.035)	87.8%	Four mild adverse events (grade 1) in the yoga group	8/10
Bland et al. (2019) [21]	Immediate exercise (n = 12) vs. Delayed exercise (n = 15)	8–12 weeks (3 sessions per week)	Moderate intensity	EORTC QLQ-CIPN20, EORTC QLQ-C30, Vibration Test, Pain Perception Test	Less progression of sensory neuropathy before the final chemotherapy cycle and greater adherence to treatment (*p* < 0.05)	78–93%	None	10/10
Cao et al. (2023) [22]	Aerobic exercise (n = 69) vs. Control (n = 65)	6 months (home-based exercise with weekly telephone supervision)	Moderate intensity	FACT/GOG-Ntx, Physical Activity Questionnaire	Significant reduction in CIPN (−1.6 points, *p* = 0.03), with no changes observed in the control group	83.8%	None	10/10
Dhawan et al. (2020) [23]	Exercise (n = 22) vs. Control (n = 23)	10 weeks (daily home-based exercise, 30 min/day)	Moderate intensity	CIPNAT, NCV, LANSS, EORTC QLQ-C30	Reduction in neuropathic pain (*p* < 0.0001) and improvement in quality of life (*p* < 0.004)	68%	None	7/10
Eroğlu & Kutlutürkan (2024) [24]	Hand and foot exercises (n = 19) vs. Control (n = 20)	8 weeks (3 sessions per day, 3 days per week)	Moderate intensity	NRS, CIPNAT, Fall Follow-Up Form, EORTC QLQ-C30, EORTC QLQ-CR29	Significant reduction in neuropathic pain and improvement in quality of life (*p* < 0.05), with no differences in fall incidence	Not reported (NR)	None	7/10
Hwang et al. (2025) [25]	Exercise app (n = 17) vs. Control with educational brochure (n = 17)	6 weeks (home-based exercise using the app)	Moderate intensity	CIPNAT, Interference with Activities Scale, EORTC QLQ-C30, Exercise Adherence Log	Fewer neuropathic symptoms (*p* = 0.002), improved quality of life (*p* = 0.003), and greater exercise adherence (*p* < 0.001)	Not reported (NR)	None	8/10
Ikio et al. (2022) [26]	Hand exercise (n = 21) vs. Control (n = 21)	6–8 weeks (3 or more sessions per week, 30 min per session)	Moderate intensity	ADL-MHQ, SWMT, Purdue Pegboard Test, VAS, PCS, FACT/GOG-Ntx, Hand Dynamometer	Less decline in ADL-MHQ (*p* = 0.0397), greater pinch strength (*p* = 0.0007), and reduced pain (*p* = 0.0083)	73.3%	None	8/10
Izgu et al. (2019) [27]	Classical massage (n = 19) vs. Control (n = 21)	12 weeks (1 session per week, 30 min)	Low to moderate intensity	S-LANSS, EORTC QLQ-CIPN20, Nerve Conduction Studies (NCS)	Reduction in neuropathic pain and improvement in quality of life (*p* < 0.05), with positive changes in nerve conduction studies	100%	None	6/10
Kneis et al. (2019) [28]	Balance + resistance training (n = 25) vs. Resistance training only (n = 25)	12 weeks (2 sessions per week)	Moderate intensity	Force Platform, Countermovement Jump (CMJ), EORTC QLQ-CIPN20, Cardiopulmonary Exercise Test (CPET)	Significant reduction in motor and autonomic symptoms, and improvement in postural control	92–100%	None	10/10
Kleckner et al. (2018) [14]	Exercise (n = 170) vs. Control (n = 185)	6 weeks (home-based walking and resistance exercise)	Moderate intensity	Neuropathy Rating Scale (0–10), Walk4Life Pedometer, Activity Log	Less neuropathy (*p* = 0.045) and improvement in sensory symptoms (*p* = 0.061)	77%	None	10/10
Joy et al. (2022) [12]	Photobiomodulation (PBM) (n = 16) vs. Placebo (n = 16)	12–18 weeks (2 sessions per week)	Class IV MLS Laser (905/808 nm, 4 J/cm^2^, 0.168 W/cm^2^)	mTNS, FACT/GOG-Taxane, 6MWT, NRS	Less symptom progression, better quality of life, reduced pain, and improved functional capacity in the PBM group	Not reported (NR)	None	9/10
Loprinzi et al. (2020) [29]	Scrambler therapy (n = 25) vs. TENS (n = 25)	2 weeks of treatment with 8-week follow-up	Not applicable (cutaneous electrical stimulation therapy)	EORTC QLQ-CIPN20, NAS (pain, tingling, numbness), Global Impression of Change	Greater reduction in neuropathic symptoms in the Scrambler group (40% vs. 20% with TENS, *p* = 0.12)	Not reported (NR)	Mild skin irritation observed in both groups	7/10
Moraitis et al. (2023) [30]	HIIT (n = 2) vs. MICE (n = 5)	12 weeks (4–5 sessions per week)	HIIT: High intensity (85–90% HRmax); MICE: Moderate intensity (50–70% HRmax)	Polar A370, Polar H10, SPPB, PROMIS Physical Function, NTSS-6, UENS, BOD POD	High adherence and improvements in quality of life, strength, and physical function, with no significant differences between groups	88.6%	None	9/10
Müller et al. (2021) [31]	Sensorimotor training (n = 52) vs. Resistance training (n = 60) vs. Control (n = 58)	20 weeks (3 sessions per week)	Moderate to high intensity (70–80% 1RM for resistance training)	TNSr, EORTC QLQ-CIPN15, EORTC QLQ-C30, COP, Isokinetic Dynamometer, FES-I, RDI	Less progression of sensory symptoms in the feet (*p* = 0.039), improved strength (*p* < 0.001), and enhanced quality of life (*p* = 0.005)	55% SMT, 49% RT	None	10/10
Saraboon & Siriphorn (2021) [32]	Balance exercise (n = 15) vs. Control (n = 15)	6 weeks (60 min, 2 sessions per week)	Moderate intensity	FAB, SPPB, MDNS, FACT-Taxane	Improved balance (*p* < 0.01), better physical function (*p* = 0.03), and higher quality of life (*p* < 0.01), with no changes in neuropathy	Not reported (NR)	None	7/10
Sassmann et al. (2024) [13]	HTEMS (n = 25) vs. TENS (n = 25) vs. Control (n = 17)	8 weeks of home-based electrotherapy treatment	Moderate intensity	EORTC QLQ-CIPN20, EORTC QLQ-C30, CTCAE v4, clinical sensory and motor examinations	Significant improvement in sensory and motor scores (EORTC QLQ-CIPN20: TENS −12.3/−8.2; HTEMS −14.7/−8.2); CIPN grade improved in both intervention groups, while physical function improved only in HTEMS (+7.9 points)	Not reported (NR)	None	6/10
Streckmann et al. (2024) [15]	SMT (n = 55) vs. WBV (n = 53) vs. Control (n = 50)	During chemotherapy (2 sessions per week, 15–30 min each)	Moderate intensity	Force platform, Rydel-Seiffer tuning fork, FACT/GOG-Ntx, EORTC QLQ-C30, Pain-DETECT	Lower incidence of CIPN (50–70%), improved balance and neuropathic pain, and reduced need for chemotherapy dose reductions	72.8%	None	10/10
Uysal & Toprak (2025) [33]	Massage ball (n = 26) vs. Stress ball (n = 26) vs. Control (n = 27)	8 weeks (daily home-based exercise)	Low to moderate intensity	NCI-CTCAE v5.0, EORTC QLQ-C30, EORTC QLQ-CIPN20	Significant reduction in CIPN (*p* < 0.001), with improvements in quality of life and reductions in pain and fatigue	>90%	None	8/10
Vollmers et al. (2018) [34]	Sensorimotor exercise (n = 17) vs. Control (n = 19)	During chemotherapy plus 6 weeks post-treatment (2 sessions per week)	Moderate intensity	Force platform, FAB, Hand Dynamometry, Chair Rising Test, EORTC QLQ-C30, BR23, CIPN20, MFI-20	Improved postural stability and prevention of strength loss (*p* < 0.001), with no significant changes in quality of life	High (not quantified)	None	6/10
Waibel et al. (2021) [35]	Balance + resistance training (n = 16) vs. Resistance training only (n = 15)	12 weeks (2 sessions per week)	Moderate intensity	Force platform, Motion Capture System, EORTC QLQ-CIPN20	Reduced postural sway, improved stability, and decreased CIPN symptoms	≥70%	None	7/10
Xiaoqian et al. (2025) [36]	Compression + exercise (n = 36) vs. Compression only (n = 36) vs. Control (n = 36)	5 chemotherapy cycles (daily exercise)	Low to moderate intensity	NCI-CTC v4.0, CIPNAT, ADL	Significant reduction in CIPN and improvement in quality of life (*p* < 0.001)	High (no dropouts)	None	7/10
Zhi et al. (2021) [37]	Yoga (n = 21) vs. Waitlist control (n = 20)	8 weeks (60 min/day, 2 in-person sessions per week)	Low to moderate intensity	HADS, BFI, ISI, TES	Reduced anxiety at 12 weeks (*p* = 0.017), with no significant improvements in fatigue, insomnia, or depression	>85%	None	6/10
Zimmer et al. (2018) [38]	Multimodal exercise (n = 17) vs. Control (n = 13)	8 weeks (2 sessions per week, 60 min each)	Moderate intensity	FACT/GOG-NTX, GGT-Reha, h1RM, 6MWT	Prevention of CIPN deterioration (*p* = 0.028), improvements in balance and strength (*p* < 0.05), with no changes in aerobic capacity	88.3%	None	10/10

## Data Availability

No new data were created or analysed in this study. Data sharing is not applicable to this article.

## Abbreviations

The following abbreviations are used in this manuscript:

6MWT	Six-Minute Walk Test
ADL-MHQ	Activities of Daily Living—Michigan Hand Outcomes Questionnaire
ASCO	American Society of Clinical Oncology
BDNF	Brain-Derived Neurotrophic Factor
BFI	Brief Fatigue Inventory
BOD POD	Air displacement plethysmography system for body composition analysis
BR23	EORTC Quality of Life Questionnaire–Breast Cancer Module
CIPN	Chemotherapy-Induced Peripheral Neuropathy
CIPNAT	Chemotherapy-Induced Peripheral Neuropathy Assessment Tool
CMJ	Countermovement Jump
COP	Centre of Pressure
CPET	Cardiopulmonary Exercise Test
CTCAE/NCI-CTCAE	Common Terminology Criteria for Adverse Events (National Cancer Institute)
DASH	Disabilities of the Arm, Shoulder and Hand questionnaire
EORTC QLQ-C30	European Organisation for Research and Treatment of Cancer Quality of Life Questionnaire–Core 30
EORTC QLQ-CIPN20/CIPN15/Taxane/CR29	EORTC Quality of Life Questionnaire–Chemotherapy-Induced Peripheral Neuropathy Module (20-, 15-item, Taxane-specific or Colorectal Cancer versions)
FAB	Fullerton Advanced Balance scale
FACT/GOG-Ntx/FACT/GOG-Taxane	Functional Assessment of Cancer Therapy/Gynaecologic Oncology Group–Neurotoxicity (or Taxane) subscale
FES-I	Falls Efficacy Scale–International
GGT-Reha	German Geriatric Rehabilitation Test battery
h1RM	Half of the one-repetition maximum strength test (50% 1RM)
HADS	Hospital Anxiety and Depression Scale
HIIT	High-Intensity Interval Training
HTEMS	High-Tone External Muscle Stimulation
ISI	Insomnia Severity Index
LANSS/S-LANSS	Leeds Assessment of Neuropathic Symptoms and Signs (Self-Reported version)
MFI-20	Multidimensional Fatigue Inventory (20-item)
MHQ	Michigan Hand Outcomes Questionnaire
MICE	Moderate-Intensity Continuous Exercise
MDNS	Modified Diabetic Neuropathy Score
MLS	Multiwave Locked System (Class IV therapeutic laser)
mTNS/TNS/TNSr	(Modified/Revised) Total Neuropathy Score
NAS	Numeric Analogue Scale (pain, tingling, numbness)
NCV/NCS	Nerve Conduction Velocity/Nerve Conduction Studies
NCI-CTC 4.0/5.0	National Cancer Institute Common Toxicity Criteria (version 4.0/5.0)
NPRS/NRS	Numeric Pain Rating Scale/Numeric Rating Scale
NTSS-6	Neuropathy Total Symptom Score–6
PCS	Pain Catastrophising Scale
PBM	Photobiomodulation
PEDro	Physiotherapy Evidence Database
PRISMA	Preferred Reporting Items for Systematic Reviews and Meta-Analyses
PROMIS Physical Function	Patient-Reported Outcomes Measurement Information System–Physical Function scale
QoL	Quality of Life
QST	Quantitative Sensory Testing
RCT	Randomised Controlled Trial
RDI	Rate of Decline Index
RT	Resistance Training
Rydel-Seiffer tuning fork	Graduated tuning fork used for vibration sensitivity testing
SPPB	Short Physical Performance Battery
SMT	Sensorimotor Training
SWMT	Semmes–Weinstein Monofilament Test
TENS	Transcutaneous Electrical Nerve Stimulation
TES	Treatment Expectancy Scale
UENS	Utah Early Neuropathy Scale
VAS	Visual Analogue Scale
WBV	Whole-Body Vibration
